# Supplementary material for: Small RNA sequencing to differentiate lung squamous cell carcinomas from metastatic lung tumors from head and neck cancers
Source: PLoS One. 2021 Mar 5;16(3):e0248206. doi: 10.1371/journal.pone.0248206 (PMC7935561; doi:10.1371/journal.pone.0248206)
Supplement: S1 Dataset — (DOCX) [file pone.0248206.s001.docx]

**Minimal data set**

**Fig 1D and 1E.**

Total of 160 known miRNAs FRKM value were measured. From these data, we performed step-wise discriminant analysis for selecting minimal miRNAs set for discriminating tumor type. Using only the selected miRNA, we obtained two discriminant functions by canonical discriminant analysis. For each case,

Linear discriminants 1 score = Σ(LD1)X (miRNA FPLM value)

Linear discriminants 2 score = Σ(LD2)X (miRNA FPLM value)

LD1/LD2: Linear discriminants 1/2 values were on Fig 1D table. Fig 1D graph was created by linear discriminants 1/2 score plotting.

| Fig. # | Mean | S.D | S.E | Statistical method used | p-value | # samples |
| --- | --- | --- | --- | --- | --- | --- |
| **Fig 2** |  |  |  | The Student *t-*test |  |  |
| miR-10a LSQCC | 13717.03 | 4087.05 | 1444.99 |  | < 0.001 | 8 |
| miR-10a MSQCC | 15.07 | 13.89 | 5.67 |  |  | 6 |
| miR-28 LSQCC | 22371.29 | 16003.04 | 5657.93 |  | 0.006 | 8 |
| miR-28 MSQCC | 17.34 | 20.06 | 8.19 |  |  | 6 |
| miR-99b LSQCC | 332.43 | 188.03 | 66.48 |  | 0.667 | 8 |
| miR-99b MSQCC | 271.4 | 329.1 | 134.36 |  |  | 6 |
| miR-141 LSQCC | 238312.8 | 187387.11 | 66251.35 |  | 0.010 | 8 |
| miR-141 MSQCC | 52.2 | 94.61 | 38.62 |  |  | 6 |
| miR-320b LSQCC | 7690.63 | 20116.44 | 7112.23 |  | 0.373 | 8 |
| miR-320b MSQCC | 4.23 | 3.93 | 1.6 |  |  | 6 |
| miR-3120 LSQCC | 4436.5 | 2893.22 | 1022.91 |  | 0.003 | 8 |
| miR-3120 MSQCC | 18.48 | 27.45 | 11.21 |  |  | 6 |
| **Fig 3** |  |  |  |  |  |  |
| miR-10a HNSQCC | 11.17 | 10.64 | 2.322 | The Student *t-*test | < 0.001 | 21 |
| miR-10a LSQCC | 47.83 | 18.96 | 4.24 |  |  | 20 |
| miR-28 HNSQCC | 5.583 | 3.945 | 0.8609 |  | < 0.001 | 21 |
| miR-28 LSQCC | 11.28 | 5.208 | 1.165 |  |  | 20 |
| miR-141 HNSQCC | 17.07 | 19.23 | 4.196 |  | 0.002 | 21 |
| miR-141 LSQCC | 52.62 | 44 | 9.838 |  |  | 20 |
| miR-3120 HNSQCC | 2.321 | 1.417 | 0.3091 |  | 0.017 | 21 |
| miR-3120 LSQCC | 3.495 | 1.587 | 0.355 |  |  | 20 |
| **Fig 4D** |  |  |  |  |  |  |
| miR-10a LSQCC-E | 46.67 | 45.62 | 16.13 | The Student *t-*test | 0.042 | 8 |
| miR-10a MSQCC-E | 6.515 | 6.261 | 2.556 |  |  | 6 |
| miR-28 LSQCC-E | 100.2 | 106.2 | 37.56 |  | 0.041 | 8 |
| miR-28 MSQCC-E | 6.327 | 6.095 | 2.488 |  |  | 6 |
| miR-141 LSQCC-E | 33.97 | 46.44 | 16.42 |  | 0.274 | 8 |
| miR-141 MSQCC-E | 11.68 | 25.65 | 10.47 |  |  | 6 |
| miR-3120 LSQCC-E | 46.5 | 51.99 | 18.38 |  | 0.047 | 8 |
| miR-3120 MSQCC-E | 2.29 | 3.2 | 1.306 |  |  | 6 |
|  |  |  |  |  |  |  |
| Fig. # | Mean | S.D | S.E | Statistical method used | p-value | # samples |
| **S1 Fig** |  |  |  |  |  |  |
| miR-10a LSQCC | 13717 | 4087 | 1445 | The Student *t-*test | < 0.001 | 8 |
| miR-10a LSQCC-E | 46.67 | 45.62 | 16.13 |  |  | 6 |
| miR-10a MSQCC | 15.07 | 13.89 | 5.672 |  | 0.212 | 8 |
| miR-10a MSQCC-E | 6.515 | 6.261 | 2.556 |  |  | 6 |
| miR-28 LSQCC | 22371 | 16003 | 5658 |  | 0.006 | 8 |
| miR-28 LSQCC-E | 100.2 | 106.2 | 37.56 |  |  | 6 |
| miR-28 MSQCC | 17.34 | 20.06 | 8.19 |  | 0.246 | 8 |
| miR-28 MSQCC-E | 6.327 | 6.095 | 2.488 |  |  | 6 |
| miR-141 LSQCC | 238313 | 187387 | 66251 |  | 0.009 | 8 |
| miR-141 LSQCC-E | 33.97 | 46.44 | 16.42 |  |  | 6 |
| miR-141 MSQCC | 52.2 | 94.61 | 38.62 |  | 0.352 | 8 |
| miR-141 MSQCC-E | 11.68 | 25.65 | 10.47 |  |  | 6 |
| miR-3120 LSQCC | 4436 | 2893 | 1023 |  | 0.004 | 8 |
| miR-3120 LSQCC-E | 46.5 | 51.99 | 18.38 |  |  | 6 |
| miR-3120 MSQCC | 18.48 | 27.45 | 11.2 |  | 0.209 | 8 |
| miR-3120 MSQCC-E | 2.29 | 3.2 | 1.306 |  |  | 6 |
