# Supplementary figures and images for: Small RNA sequencing to differentiate lung squamous cell carcinomas from metastatic lung tumors from head and neck cancers
Source: PLoS One. 2021 Mar 5;16(3):e0248206. doi: 10.1371/journal.pone.0248206 (PMC7935561; doi:10.1371/journal.pone.0248206)

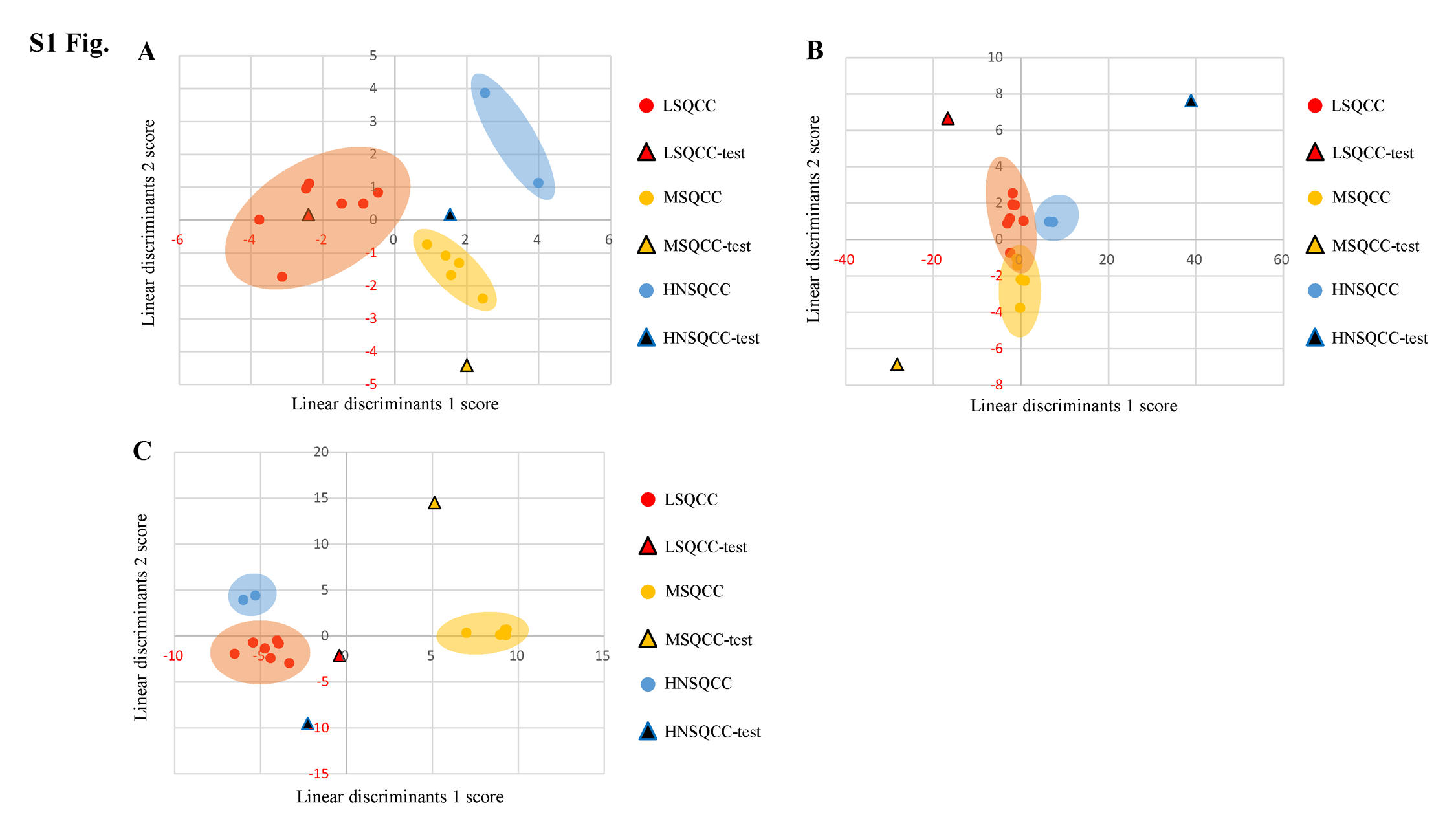

Supplement: S1 Fig — Each different classification with linear discriminant analysis with the coefficients of each miRNA’s linear discriminants achieved the good separation of each histologic type (A, B, and C). Type I and II errors in the validation set were 11% and 1%, respectively. An error rate obtained by the random forest algorithm was 5.88%. (TIF) [file pone.0248206.s002.tif]

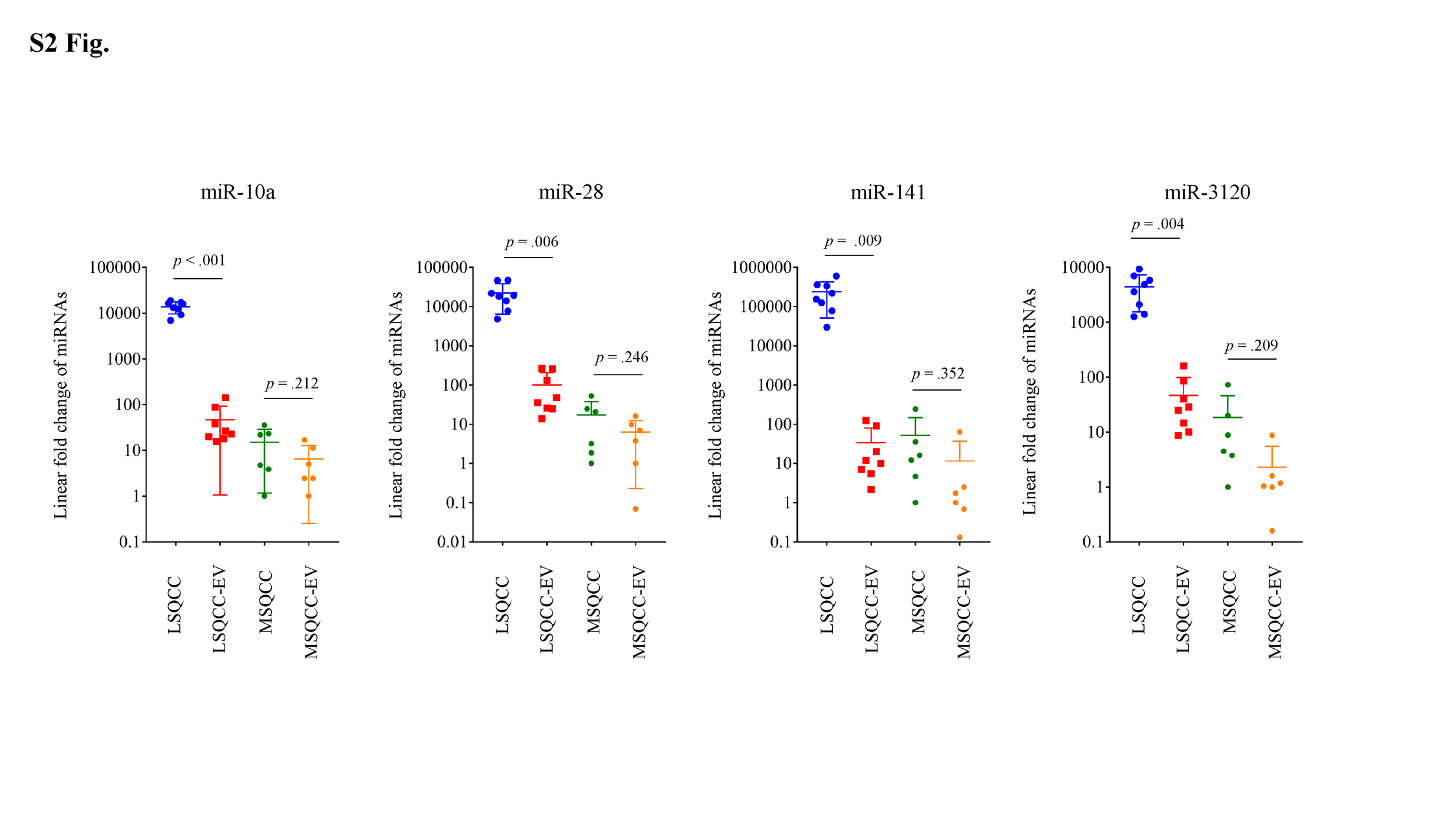

Supplement: S2 Fig — Formalin-fixed paraffin-embedded samples from LSQCC patients had significantly higher miR-10a (p < .001), miR-28 (p = .006), miR-141 (p = .009), and miR-3120 (p = .004) levels than serum extracellular vesicle samples from the matched patients. (TIF) [file pone.0248206.s003.tif]

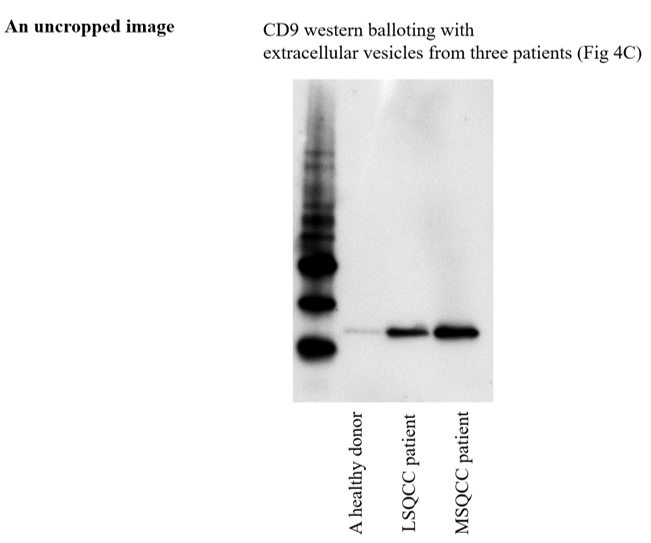

Supplement: S1 Raw image — (TIF) [file pone.0248206.s004.tif]
