# Supplementary material for: Small RNA sequencing to differentiate lung squamous cell carcinomas from metastatic lung tumors from head and neck cancers
Source: PLoS One. 2021 Mar 5;16(3):e0248206. doi: 10.1371/journal.pone.0248206 (PMC7935561; doi:10.1371/journal.pone.0248206)
Supplement: Attachment — Submitted filename: Response to Reviewers.doc [file pone.0248206.s005.doc]

October 26, 2020

Joerg Heber

Editor-in-Chief
*PLOS ONE*

Sumitra Deb

Academic editor

*PLOS ONE*

Manuscript ID PONE-D-20-26356 entitled **"Small RNA sequencing to differentiate lung squamous cell carcinomas from metastatic lung tumors from head and neck cancers"**

Dear Dr. Heber, Deb

Thank you so much for your letter of 2-October-2020, stating your comments to our manuscript. We have carefully revised our manuscript and responded all the comments from reviewers as follows.

**Academic Editor**

**-COMMENT 1**

Please ensure that your manuscript meets PLOS ONE's style requirements, including those for file naming. The PLOS ONE style templates can be found at <https://journals.plos.org/plosone/s/file?id=wjVg/PLOSOne_formatting_sample_main_body.pdf> and <https://journals.plos.org/plosone/s/file?id=ba62/PLOSOne_formatting_sample_title_authors_affiliations.pdf>

-ANSWER to comment 1: Thank you for this notification. We rechecked our manuscript following the style requirements.

**-COMMENT 2**

Please provide additional details regarding participant consent. In the ethics statement in the Methods and online submission information, please ensure that you have specified what type you obtained (for instance, written or verbal, and if verbal, how it was documented and witnessed). If your study included minors, state whether you obtained consent from parents or guardians. If the need for consent was waived by the ethics committee, please include this information.

-ANSWER to comment 2: Revised description is shown in Methods accordingly.

-CHANGES: Written informed consent for the use and analyses of clinical data was obtained preoperatively from each patient. (Page 9, Line 6)

**-COMMENT 3**

Thank you for stating in your Funding Statement:

"This study was supported in part by a Grant-in-Aid for Cancer Research from the Ministry of Education, Culture, Sports, and Technology, Japan (#18K08799; Y. Shimada)."

Please provide an amended statement that declares *all* the funding or sources of support (whether external or internal to your organization) received during this study, as detailed online in our guide for authors at <http://journals.plos.org/plosone/s/submit-now>. Please also include the statement “There was no additional external funding received for this study.” in your updated Funding Statement.

Please include your amended Funding Statement within your cover letter. We will change the online submission form on your behalf.

-ANSWER to comment 3: According to the comment, we revised the funding statement as follows.

-CHANGES: This study was supported in part by a Grant-in-Aid for Cancer Research from the Ministry of Education, Culture, Sports, and Technology, Japan (#18K08799; Y. Shimada), and internal research funding from department of thoracic surgery, Tokyo Medical University. There was no additional external funding received for this study. (Page 2, Line 13)

**-COMMENT 4**

In your Data Availability statement, you have not specified where the minimal data set underlying the results described in your manuscript can be found. PLOS defines a study's minimal data set as the underlying data used to reach the conclusions drawn in the manuscript and any additional data required to replicate the reported study findings in their entirety. All PLOS journals require that the minimal data set be made fully available. For more information about our data policy, please see <http://journals.plos.org/plosone/s/data-availability>.

-ANSWER to comment 4: According to this requirement, we create a minimal data set document.

**-COMMENT 5**

PLOS ONE now requires that authors provide the original uncropped and unadjusted images underlying all blot or gel results reported in a submission’s figures or Supporting Information files. This policy and the journal’s other requirements for blot/gel reporting and figure preparation are described in detail at [https://journals.plos.org/plosone/s/figures#loc-blot-and-gel-reporting-requirements](https://journals.plos.org/plosone/s/figures#loc-blot-and-gel-reporting-requirements) and [https://journals.plos.org/plosone/s/figures#loc-preparing-figures-from-image-files](https://journals.plos.org/plosone/s/figures#loc-preparing-figures-from-image-files). When you submit your revised manuscript, please ensure that your figures adhere fully to these guidelines and provide the original underlying images for all blot or gel data reported in your submission. See the following link for instructions on providing the original image data: [https://journals.plos.org/plosone/s/figures#loc-original-images-for-blots-and-gels](https://journals.plos.org/plosone/s/figures#loc-original-images-for-blots-and-gels).

In your cover letter, please note whether your blot/gel image data are in Supporting Information or posted at a public data repository, provide the repository URL if relevant, and provide specific details as to which raw blot/gel images, if any, are not available. Email us at [plosone@plos.org](mailto:plosone@plos.org) if you have any questions.

-ANSWER to comment 5: According to the comment, we created a figure with an uncropped image of western blotting as follows.

-CHANGES:

**-COMMENT 6**

PLOS requires an ORCID iD for the corresponding author in Editorial Manager on papers submitted after December 6th, 2016. Please ensure that you have an ORCID iD and that it is validated in Editorial Manager. To do this, go to ‘Update my Information’ (in the upper left-hand corner of the main menu), and click on the Fetch/Validate link next to the ORCID field. This will take you to the ORCID site and allow you to create a new iD or authenticate a pre-existing iD in Editorial Manager. Please see the following video for instructions on linking an ORCID iD to your Editorial Manager account: <https://www.youtube.com/watch?v=_xcclfuvtxQ>

-ANSWER to comment 6: According to this, my ORCID iD was registered.

**Reviewer’s comments**

**Reviewer #1**

**-COMMENT 1**

For consistency if data in Fig 2 and Fig 4 D is presented as a scatter dot plot as done in in Fig 3 will be more informative.

-ANSWER to comment 1: Thank you for this advice. According to the comment, we revised figures as follows.

-CHANGES:

Figure 4D

**-COMMENT 2**

It is unclear if all the isolated vesicles were characterized by nanoparticle characterizing system or just some. Is Figure 4B. just a representative of sample from one patient?

-ANSWER to comment 2: Thank you for this comment. That is a representative of samples from one patient. We have not performed nanoparticle trafficking analysis (NTA) for all patients. As we have shown, an exosome isolation kit with membrane protein magnetic beads was used in this project, and we confirmed the identification of many small vesicles from a total of 4 patients whose size is around 100 nm in diameter in samples by NTA and electron microscope. We added the phrase as follows.

-CHANGES:

We checked the identification of extracellular vesicles to ensure consistency of the results in a total of 4 patients. (Page 14, Line 4)

**-COMMENT 3**

Have the authors compared the miRNA levels in matched FFPE and serum samples? If so, it would be informative have it as supplementary data.

-ANSWER to comment 3: Thank you for this important advice. We can show results for comparison of gene expression from two different sources. Supplementary figure 1 is added as follows.

-CHANGES:

**-COMMENT 4**

Some sections if re-written or re-phrased will improve the flow and readability of the manuscript.
a) Discussion section: Page 16, last paragraph, first two sentences read the same and have the same conclusion.
b) Discussion section: Page 18. 2nd paragraph- “miRNA profiles of 13 miRNAs could identify the 3 cancer groups as having epigenetically distinct manifestations.” It is not very clear what the author is trying say.

-ANSWER to comment 4: Thank you for checking out these points. According to the first comment, we revised the sentence as follows. We also revised the second issue for more readable as follows.

-CHANGES:

a) The validation dataset with FFPE from MSQCC and LSQCC demonstrated that miR-10a, miR-28, miR-141, and miR-3120 were significantly higher in LSQCCs than MSQCCs, while the other dataset revealed that these 4 miRNAs were more abundant in LSQCCs than HNSQCCs. The expression levels of miR-10a, miR-28, and miR-3120 in serum extracellular vesicles from LSQCC patients were significantly higher than those from MSQCC patients. (Page 17, Line 3)

b) Although LSQCC and MSQCC samples in the derivation set from patients diagnosed based on pathological review had the potential to yield biased results, miRNA profiles of 13 miRNAs could classify all the samples into 3 groups, LSQCC, MSQCC, HNSQCC, according to epigenetic expression. (Page 18, Line 18)

**Reviewer #2**

**-COMMENT 1**

It would help the average reader to have an explanation of how to interpret the coefficients from the discriminant analysis models.

-ANSWER to comment 1: Thank you for this important advice. According to the comment, we added the following phrase.

-CHANGES:

The absolute values of coefficients correspond to variables with more exceptional discriminant ability. The coefficient values meaning indicates how much a given miRNA contributed to a discriminant function. (Page 12, Line 11)

**COMMENT 2**

This study shows the development and use of the models, but does not test the models for robustness or assess the potential of over-fitting. This would be done by cross-validation such as with Leave-One-Out based on the small sample size. The Type I and Type II errors and typical metrics for evaluating models, such as sensitivity and specificity, would then be presented. Results from model validation were not presented and models cannot be evaluated without this validation.

-ANSWER to comment 2: Thank you for this critical point. For determining potential grouping of miRNA distinguishing three types of SQCC in this study, we performed a stepwise discriminant analysis by using known miRNAs. It might have been more understandable if we could test our discriminant model to ensure robustness and to assess the potential of over-fitting with the other different SQCC datasets or collect more SQCC cases before the initial small-RNA sequencing. However, because MSQCC is a relatively rare form of lung tumors, we could collect only 13 MSQCC cases and had to separate those samples into a training cohort and a validation cohort. Linear discriminant analysis is a method to evaluate how well a group of variables supports a priori grouping objects. We finally found significant differences of epigenetic expressions among the 3 groups by using a total of 17 SQCC samples used for small RNA-sequencing followed by the discriminant analysis (Fig 1). The model was created for just selecting the variable miRNAs set for tumor type discrimination. The selected miRNAs were tested and verified by following qRT-PCR process. Thirteen miRNAs were identified as specific variables with high discriminatory power. Because this single discriminant analysis enabled us to find out significant miRNAs for practical and mathematically analytical viewpoints, we conclude that there is no need for cross-validation of the stepwise discriminant analysis in this setting. I appreciate that the reviewer pointed it out and provided us with better opportunities to assess our model again.

The comments offered by the reviewers and editors have been helpful in formulating what we believe is a stronger paper. We appreciate these thoughtful comments, and hope that our manuscript is now suitable for publication in *PLOS ONE.*

All related correspondence should be sent to Yoshihisa Shimada, M.D., Ph.D.

Department of Surgery, Tokyo Medical University Hospital

6-7-1 Nishishinjuku, Shinjyuku-ku, Tokyo, 160-0023, Japan

Phone: +81-(0)3-3342-6111, Fax: +81-(0)3-3342-6203

E-male: [zenkyu@za3.so-net.ne.jp](mailto:zenkyu@za3.so-net.ne.jp)

Sincerely yours,

Yoshihisa Shimada, M.D., Ph.D.
