# Supplementary material for: Small RNA sequencing to differentiate lung squamous cell carcinomas from metastatic lung tumors from head and neck cancers
Source: PLoS One. 2021 Mar 5;16(3):e0248206. doi: 10.1371/journal.pone.0248206 (PMC7935561; doi:10.1371/journal.pone.0248206)
Supplement: Attachment — Submitted filename: Response to Reviewers.doc [file pone.0248206.s006.doc]

December 5, 2020

Joerg Heber

Editor-in-Chief
*PLOS ONE*

Sumitra Deb

Academic editor

*PLOS ONE*

Manuscript ID PONE-D-20-26356R1 entitled **"Small RNA sequencing to differentiate lung squamous cell carcinomas from metastatic lung tumors from head and neck cancers"**

Dear Dr. Heber, Deb

Thank you so much for your letter of 24-November-2020, stating your comments to our manuscript. We have carefully revised our manuscript and responded all the comments from reviewers as follows.

**Reviewer #2**

**-COMMENT 1**

This paper indicates that it shows differentiation between LSQCC and MSQCC using miRNAs. The title says miRNAs are used to differentiate between LSQCC and MSQCC, the Abstract and manuscript text says that the aim is to determine if miRNAs expression can be used to differentially diagnose LSQCC and MSQCC, and it’s concluded that miRNA sequencing and quantitation is useful for differential diagnosis of LSQCC and MSQCC. However, no evidence was presented that shows how well any single miRNA or combination differentiated between LSQCC and MSQCC. This problem was stated in the first review and it continues to be true in this authors’ revision. As stated in the first review, some form of metrics is required to assess if the miRNAs are capable of differentiating between LSQCC and MSQCC and thus might be useful for diagnosis. Without any assessment of how well or how poorly miRNA expression can be used to distinguish between LSQCC and MSQCC, the above-mentioned statements by the authors are incorrect. What this paper does show is the differential expression of miRNAs between LSQCC and MSQCC. If the authors restrict the paper to this topic, the paper is fine. Speculation that the identified miRNAs might be useful for distinguishing between LSQCC and MSQCC is fine.

-ANSWER to comment 1: Thank you for this critical comment. What the reviewer pointed out is totally true. The validation is required to assess if the miRNAs can differentiate MSQCC from LSQCC with different datasets. It is supposed to be better that the versatility of our model is evaluated with the other set of samples. However, we cannot perform the same dimensional validation because the current version of miRNA kit probe is different from the previous version of it that was used as our model. Therefore, we randomly extracted 1 case (LSQCC-test, MSQCC-test, and HNSQCC-test) each from the 3 groups as the validation case, and the rest 14 cases were used to generate the new 13-miRNA-based models. We added the following phrase and supplementary figure as follows.

-CHANGES: To evaluate the performance of the model with the miRNAs to distinguish LSQCC from MSQCC and HNSQCC, 1 case (LSQCC-test, MSQCC-test, and HNSQCC-test) was randomly extracted from each group as the validation case, and the rest 14 cases were used to generate the new 13-miRNA-based models. Three-fold cross-validation identified that the miRNAs were capable of differentiating LSQCC and MSQCC (S1 Fig A, B, C). (Page 15, Line 18)

The comments offered by the reviewers and editors have been helpful in formulating what we believe is a stronger paper. We appreciate these thoughtful comments, and hope that our manuscript is now suitable for publication in *PLOS ONE.*

All related correspondence should be sent to Yoshihisa Shimada, M.D., Ph.D.

Department of Surgery, Tokyo Medical University Hospital

6-7-1 Nishishinjuku, Shinjyuku-ku, Tokyo, 160-0023, Japan

Phone: +81-(0)3-3342-6111, Fax: +81-(0)3-3342-6203

E-male: [zenkyu@za3.so-net.ne.jp](mailto:zenkyu@za3.so-net.ne.jp)

Sincerely yours,

Yoshihisa Shimada, M.D., Ph.D.
