# Supplementary material for: Small RNA sequencing to differentiate lung squamous cell carcinomas from metastatic lung tumors from head and neck cancers
Source: PLoS One. 2021 Mar 5;16(3):e0248206. doi: 10.1371/journal.pone.0248206 (PMC7935561; doi:10.1371/journal.pone.0248206)
Supplement: Attachment — Submitted filename: Response to Reviewers.doc [file pone.0248206.s007.doc]

December 15, 2020

Joerg Heber

Editor-in-Chief
*PLOS ONE*

Sumitra Deb

Academic editor

*PLOS ONE*

Manuscript ID PONE-D-20-26356R2 entitled **"Small RNA sequencing to differentiate lung squamous cell carcinomas from metastatic lung tumors from head and neck cancers"**

Dear Dr. Heber, Deb

Thank you so much for your letter of 8-December-2020, stating your comments to our manuscript. We have carefully revised our manuscript and responded all the comments from reviewers as follows.

**Reviewer #2**

**-COMMENT 1**

The cross-validation is limited. All samples must be validated to avoid bias.
Readers should not have to "eyeball" the results of the validation to ascertain how well the model performed. The performance of the model should be quantitated by Type I and Type II errors.

-ANSWER to comment 1: Thank you for this comment. Because the validation would deviate from the main line of the study, we described it in supplementary figure legends as follows.

-CHANGES: **S1 Fig. Three-fold cross-validation of our model with the 13 miRNAs**. Each different classification with linear discriminant analysis with the coefficients of each miRNA’s linear discriminants achieved the good separation of each histologic type (A, B, and C). Type I and II errors in the validation set were 11% and 1%, respectively. An error rate obtained by the random forest algorithm was 5.88%.

The comments offered by the reviewers and editors have been helpful in formulating what we believe is a stronger paper. We appreciate these thoughtful comments, and hope that our manuscript is now suitable for publication in *PLOS ONE.*

All related correspondence should be sent to Yoshihisa Shimada, M.D., Ph.D.

Department of Surgery, Tokyo Medical University Hospital

6-7-1 Nishishinjuku, Shinjyuku-ku, Tokyo, 160-0023, Japan

Phone: +81-(0)3-3342-6111, Fax: +81-(0)3-3342-6203

E-male: [zenkyu@za3.so-net.ne.jp](mailto:zenkyu@za3.so-net.ne.jp)

Sincerely yours,

Yoshihisa Shimada, M.D., Ph.D.
