# Supplementary material for: Small RNA sequencing to differentiate lung squamous cell carcinomas from metastatic lung tumors from head and neck cancers
Source: PLoS One. 2021 Mar 5;16(3):e0248206. doi: 10.1371/journal.pone.0248206 (PMC7935561; doi:10.1371/journal.pone.0248206)
Supplement: Attachment — Submitted filename: Response to Reviewers.doc [file pone.0248206.s008.doc]

January 27, 2021

Joerg Heber

Editor-in-Chief
*PLOS ONE*

Sumitra Deb

Academic editor

*PLOS ONE*

Manuscript ID PONE-D-20-26356R3 entitled **"Small RNA sequencing to differentiate lung squamous cell carcinomas from metastatic lung tumors from head and neck cancers"**

Dear Dr. Heber, Deb

Thank you so much for your letter of 30-December-2020, stating your comments to our manuscript. We have carefully revised our manuscript and responded all the comments from reviewers as follows.

**Reviewer #3**

The authors need to provide statistical analysis for Fig 5b. Also since the cell lines used in Fig 5 are already established providing an image for cells as shown in 5a seems redundant. Instead authors may analyze more cell lines to strengthen their conclusion.

Answer:

Thank you for your advice. We set up this in vitro analysis with several cell lines (3 lung cancers and 1 head and neck cancer) again. The miR3120 level in LSQCCs was significantly higher than that in the HNSQCC line, whereas the remaining gene levels were not significantly different between the 2 histological types. There is no consistency in the results by patient’s sample experiments and in vivo ones. Therefore, we exclude Figure 5 form our article. The following phrase is added.

Change:

We checked the levels of the 4 miRNAs using extracellular vesicles derived from 4 LSQCC cell lines (EBA-1, H520, and LK-2 purchased from ATCC) and 1 HNSQCC cell line (BHY purchased from DSMZ) to examine whether the gene expressions in patients’ samples agree with those in cell lines. There were no significant differences in the expressions between two subtypes except for miR-3120 (data not shown).

The comments offered by the reviewers and editors have been helpful in formulating what we believe is a stronger paper. We appreciate these thoughtful comments, and hope that our manuscript is now suitable for publication in *PLOS ONE.*

All related correspondence should be sent to Yoshihisa Shimada, M.D., Ph.D.

Department of Surgery, Tokyo Medical University Hospital

6-7-1 Nishishinjuku, Shinjyuku-ku, Tokyo, 160-0023, Japan

Phone: +81-(0)3-3342-6111, Fax: +81-(0)3-3342-6203

E-male: [zenkyu@za3.so-net.ne.jp](mailto:zenkyu@za3.so-net.ne.jp)

Sincerely yours,

Yoshihisa Shimada, M.D., Ph.D.
